# Continuous quality improvement in measure development: Lessons from building a novel clinical feedback system

**DOI:** 10.1007/s11136-021-02768-7

**Published:** 2021-02-16

**Authors:** Samuel S. Nordberg, Andrew A. McAleavey, Christian Moltu

**Affiliations:** 1 Harvard School of Population Medicine, Boston, MA USA; 2 Weill Cornell Medical School, New York, NY USA; 3 District General Hospital of Førde, Førde, Norway; 4 Department of Health and Caring Sciences, Western Norway University of Applied Science, Førde, Norway

**Keywords:** Clinical feedback system, Routine outcome monitoring, Continuous quality improvement

## Abstract

**Purpose:**

While the use of clinical feedback systems has become commonplace in psychological treatment, many of the most common instruments used for this purpose have not changed in decades. This paper describes the first four cycles of a measure development method designed to embrace continuous quality improvement.

**Methods:**

Using techniques and philosophies developed in business management and academia—lean continuous quality improvement, action research, and practice research networks—we iterated through multiple cycles of development with the goal of creating an optimal clinical feedback system. These cycles emphasize building capacity to receive and implement feedback from a variety of stakeholders, especially patients and providers of behavioral health services, while also being responsive to quantitative findings from measure development.

**Results:**

Iterating measure development with stakeholder feedback over the course of 5 years has resulted in a novel measurement system with 19 subscales administered via branching logic, and a supporting practice research network to sustain development.

**Conclusion:**

In developing a new clinical feedback system, the less-frequently-discussed practical aspects of measure development require close attention. Specifically, being willing to embrace change, planning for iteration, and systematically seeking stakeholder feedback are identified as central methods for improving clinical feedback systems.

## Introduction

One of the core principles of measure development is continuous evaluation and improvement [e.g. 1–4]. We have proposed that clinical feedback systems (CFS), now widely used in mental health and substance use, have largely ignored continuous ongoing development, and have pointed out that the most widely used CFS measures have not changed for over a decade, despite the opportunity to learn and improve from feedback of their own—from their users [5]. This is particularly important for CFS rather than other measurement tasks, as treatments change over time, new trainees enter the field, and systems of healthcare delivery change rapidly. Stagnant measures therefore not only threaten the utility of their instruments, but also endanger patients whose care depends on data from these sources. Moreover, this ignores the fact that patients frequently report that their concerns are poorly represented by standardized questionnaires [6].

Cronbach and Paul Meehl [7] point to this critical challenge for CFS developers: “One does not validate a test, but only a principle for making inferences” (p. 297). In other words, any CFS is valid for a set of explicit purposes or utilities, which are not abstract concepts, but living, breathing, changing, demands from those who use a system. Developers of such a system must regularly re-evaluate the driving purpose behind a CFS, and whether their particular system is valid for that purpose, or they risk loss of utility. Stakeholders—those who are actively involved with and depend on a system functioning well—must be consulted, and regularly. In this paper, we describe the first four iterations of a continuous quality improvement (CQI) process as applied to a new CFS: Norse Feedback (NF). The purpose of this method has been to directly tie CFS validity to stakeholder feedback—particularly from patients and providers.

This manuscript is a companion to quantitative and qualitative descriptions of the development, reliability and validity of the NF measure that emerged over these cycles. Our goal is to present the underlying theory, strategy, and process decisions that guided the development of this measure. These are the types of non-psychometric factors that greatly influence the process of measurement and feedback, and are therefore worth reporting. As such, we report the decisions that led to each stage of development, and describe how data and feeedback influenced these decisions, without focusing on those results. We briefly describe each phase of the development, which is now in its fifth iteration.

## Method

The method for development involves the combination of several different methodologies: lean CQI [e.g. 8, 9], action research [e.g. 10] and a practice-research network [PRN; 11] approach. Lean CQI is a business-oriented method for systematic improvement that has been applied to routine health care settings. Action research is a strategy that involves participants in the development of empirically based solutions to problems they experience. PRNs are collaborative research-clinical groups that involve clinicians in the development of research studies at each stage, and that conduct research in naturalistic settings. Together, these approaches offer methods for iteratively evolving a clinical feedback system within the same environment for which it is designed, so that it remains relevant and sensitive to the needs of users. We briefly describe the key features of this method below.

Central to the lean approach are stringent methods for *continuous* iterative development through stakeholder feedback integration. Iterative development is well documented in lean methodology, through standardized phases of *Plan, Do, Study, Act* [8]. When applied in a clinical setting, these steps conceptually overlap with intervention testing in action research, and with PRN methods, in that they require clear questions and expectations, are data driven, and focus on integrating the needs of the stakeholders in a system. For example, the *Plan* phase involves collecting comprehensive data about a problem and considering how to address it—including developing hypotheses about how participants will respond to an experimental solution. Critical to the method is the need to collect input from representatives of every group impacted by the system and proposed changes. Next, in the *Do* phase, an experiment is conducted. The process is changed deliberately and systematically, based on the hypotheses from the *Plan* phase. This process runs in a naturalistic environment, (such as a PRN), while data are collected. The *Study* phase, then, consists of an evaluation of the collected data, as well as stakeholder feedback, and a determination as to whether or not the hypothesized changes have taken place. Lastly, in the *Act* phase, a decision is made about whether and how to deploy the experiment across entire systems. This method differs from typical research by directly initiating a process of implementation and dissemination throughout a system, thereby providing an impetus to resume the process at the *Plan* stage again as new challenges are encountered. In our implementation, this approach facilitated highly clinically-focused research, targeted at solving problems encountered by clinicians and patients.

### Development narrative

#### First PDSA cycle (April 2014–April 2015)

##### Plan: initial preparations

In lean methodology, an oversight body, committed to the method, is required to make decisions, set timelines, and own the CQI process. This oversight group was first formed to explore the development of a novel measurement system in April 2014 and consisted of the first and last authors, expanding through subsequent phases.

First, we worked to build a PRN with the capacity to meet data collection and clinical testing needs. We established collaboration within an organization that already operated on a centralized digital platform [12], had strong senior leadership buy-in for clinical data collection, and sophisticated digital security infrastructure already in place (the Health West service region of Norway). This gave us access to a PRN that was large enough to power our initial research, easy to work with, and accepting of a continuous improvement process. We identified an unaffiliated measurement software partner that had already successfully negotiated security details with the Health West region and was offering various standardized assessments across medical specialties. Lastly, we approached the Regional Committees for Medical and Health Research Ethics (REC) and the project was determined exempt by the REC (2018/993/REK nord) from the Act on medical and health research and conducted in accordance with local institutional Data Protection Officer.

We continued the first *Plan* phase with an assessment of the needs of key stakeholders: patients and providers. We identified two additional stakeholder groups—researchers and administrators—but determined to put off addressing the needs of the latter, and presumed that the first and last (and, ultimately, second) authors could represent the former [13]. As we set about to understand what stakeholder groups wanted from a CFS, we assumed that some of their feedback would conflict. This was, we believed, critically important to making a more relevant CFS. Innovation would be found in navigating conflicting needs that had not been addressed previously.

Sample characteristics for each cycle are included in Table 1. Eight focus groups and five individual interviews were conducted with patients (*n* = 18) and clinicians (*n* = 37), to give us a sense of what kind of inferences needed to be drawn from a CFS [14], and what stakeholders needed from such a system. In essence—for which purposes were we trying to develop a valid measure? The methods and results of these groups are reported elsewhere [13], and we summarize the critical findings here. Participating patients were recruited through written letters explaining the purpose and design of the study, as well as through verbal information and invitation through their therapists. Patients who volunteered could elect either to participate in a focus group (*n* = 13) or individual interview (*n* = 5).

**Table 1:** Phases and associated subject samples

Cycle: study	*N*	Sex	Characteristics
Cycle 1: stakeholder interviews (patients)	18	39% male	Years of experience as a patient: - 7 (6–10) - 6 (11–20) - 4 (21–30) - 1 (31–40) Proportion inpatient vs. outpatient: - 38% inpatient, 62% outpatient
Cycle 1: stakeholder interviews (clinicians)	37	32% male	Years in practice: - 3 (< 5 years) - 19 (6–15 years) - 15 (16–30 years)
Cycle 1: initial factor structure	550	26% male	98% Heterosexual Level of Education: - 102 No University - 167 1–3 Years University - 168 University - 123 Masters - 14 Doctoral 23 in Current Mental Health Treatment
Cycle 2: branching logic design and testing And Cycle 3: re-evaluating items and measure structure	794	22% male	98% Heterosexual Level of Education: - 149 No University - 223 1–3 Years University - 222 University - 170 Masters - 30 Doctoral 36 in Current Mental Health Treatment
Cycle 3: re-evaluating items and measure structure (clinical sample)	222	33% male	96% Heterosexual Level of Education: - 113 No University - 39 Vocational School - 48 University - 6 Masters Clinical Setting for data collection: - 41 Inpatient - 171 Outpatient
Cycle 4: re-evaluating dynamic structure	323	30% male	No other demographics provided for this clinical sample

Qualitative analysis of provider and patient interviews [13], indicated that preferred feedback topics were those which patients themselves typically think about and discuss, in their own terms, especially relationship issues and functional abilities. In short, patients wanted measurement, feedback, and communication to take place at a level consistent with the patient’s lived experience. As researchers, this gave us some pause—we also wanted a measure that was not so idiosyncratic that it could not be used for good and generalizable research. As clinicians, we were confident we could generate the idiosyncratic information necessary to tailor treatment to a particular patient. What clinicians in our study wanted to know was “how severe is this patient relative to others?” That could only be done through standardized measures. This conflict gave rise to the first critical decision in developing NF. Instead of creating a depression subscale, for example, we determined to attempt to separately identify phenomenological constituents like sad affect, self-criticism, sleep, hopelessness, social support, and resilience, which would fulfil the focus on lived experience. Each phenomenon would get its own brief scale. These constituent “subscales” could apply across a wide range of diagnostic constructs (physiological anxiety is present in GAD, PTSD, Panic Disorder, etc.) and would later be used for higher-level analyses done at the research and administration levels to detect whether higher order patterns in discretely measured phenomena resulted in constructs or patterns of elevation (which might correspond with diagnoses). The higher-order constructs could then become a critical part of the feedback to the clinician, while letting the interface of the system focus on lived experiences, to accommodate the needs of patients.

##### Do: initial item development

The process of item pool development, from a psychometric perspective, is covered in a separate manuscript [15]. Briefly, initial items were developed to focus on the lived experiences most emphasized by patients. The process specifically involved two clinical psychologists (Author initials omitted for blind review), using purposive member-checking with select patients and clinicians, and employing a lean facilitation method to brainstorm an overall approach to asking questions (i.e., item stem, tense, etc.), the targets of measurement, and, potential item formulation. This process resulted in 90 total items: 81 preliminary consensus subscale-items, as well as 5 items related to the therapeutic alliance, and 4 items to allow patients to provide feedback on their experience with their clinician. These are summarized in Table 2.

**Table 2:** NORSE 1.0 to 2.0 scale changes

NORSE 1.0 Scale	Items	Norse 2.0 Scale	Items
Attachment	4	–	–
Avoidance	6	Situational avoidance	3
–	–	Social avoidance	3
Connectedness	6	Social safety	6
Demoralization	5	Hopelessness	5
Eating problems	6	Eating problems	5
Emotional distancing	2	Internal avoidance	5
Hurtful rumination	5	Worry	3
Hypervigilance	3	Trauma reaction	4
Perfectionism-control	6	Control	4
Pressure from negative affect	6	Sad affect	4
Psychosis	3	–	–
Relational distress	5	Irritability	3
Resilience and personal coping	10	General functioning	3
Social role functioning	2		
Somatic anxiety	2	Somatic anxiety	5
Substance use	4	Substance use	4
–	-	Substance recovery	4
Suicide risk	4	Suicide risk	4
-		Self-criticism	7
-		Cognitive problems	6
-		Readiness for recovery	3
-		Recovery environment	5
Scale items	79	Branching-Logic Scale items	86
Single item	2	Single Item	5
Alliance	4	Alliance	4
Expressed needs in therapy	4	Expressed needs in therapy	5
Medication needs	2	Medication needs	2
Total items	91	Total items	102

##### Study: measure structure and utility

We then deployed the assessment on paper, to 550 anonymous research subjects who were students and professionals working and living near the PRN location, and evaluated the factor structure and item performance via principle components analysis. We fed this information back to patients and providers within the PRN, and collected their feedback for one final iteration. Based on this, we determined that several critical phenomena were missing from the assessment and returned to patients for their input on candidate items for those scales, ultimately adding additional scale-items and single-items to fill in those gaps (Table 2). We termed this, NF 1.0.

##### Act: initial measure finalization and deployment

An initial 17 subscale structure was finalized for deployment and testing in collaboration with the clinical site partner. It did not have established psychometric properties, but it did begin to capture those domains endorsed as most important to our stakeholders. We partnered with a Norwegian measurement delivery software company who had already met the data-security requirements for use in our PRN. The first and last author facilitated brainstorming sessions with programmers from the vendor to develop an initial feedback format that would appeal to clinicians and be shareable with patients. We determined that the measure was not yet ready for widespread clinical deployment and moved on to the second PDSA cycle with a new target for improvement.

#### Second PDSA cycle (May 2015–August 2015)

##### Plan: dynamic assessment methodology

As part of the data collection process in the first PDSA cycle, we learned that the measure had required approximately 15 min to complete for subjects from Cycle 1. This presented a challenge regarding typical barriers to use, such as time-commitment and relevance [16, 17]. We knew that we needed to measure broadly, to increase the likelihood of asking relevant questions to each patient, but we also knew that 15 min was too long—particularly for repeated use. We also knew that for any given patient many of the scales did not apply (e.g. Substance Use, Psychosis, Suicide). Thus, we determined to develop and test a dynamic approach to measurement that would continue to meet the need for complex assessment while also shortening the assessment time significantly by eliminating those subscales that were not relevant to a given patient. Critically, at this decision-point, we accepted that the measure would only be deliverable digitally due to its dependence on logic and algorithms. We deemed this a worthwhile trade-off—we could measure broadly across a wide array of clinical phenomena, but each patient would only repeatedly complete those scales which were relevant (i.e. elevated) for them.

**Fig. 1:**
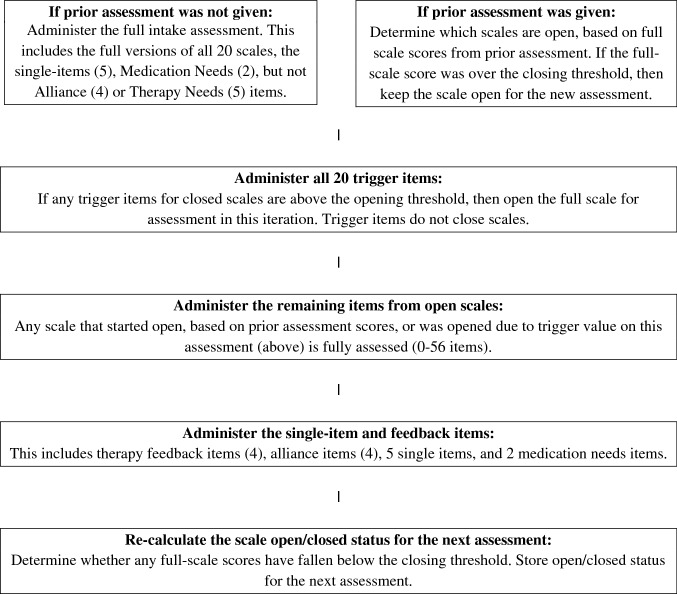
Norse feedback branching logic

Both therapists and patients had stated that tracking individually relevant items was important. Therefore, we decided we would need to develop a new branching logic method for adapting the NF, in which subscales were either “open” with all items asked, or “closed” with some means of re-opening if the scale became relevant at a later date. The branching logic structure is laid out in Fig. 1.

##### Do: measurement changes

We determined closing “thresholds”, below which a subscale would “close” if the running mean of the last 3 assessments fell below the threshold. When a subscale was closed, only one “trigger” item from the subscale would be asked. Each trigger also had a threshold; scores on the trigger item above that threshold would indicate worsening symptoms, and trigger re-opening the full scale. Thus, scales could open or close multiple times throughout a treatment. Initial triggers were those with the highest correlations with the other items on the same scale.

##### Study: adaptation evaluation

We administered the full assessment to a sample of anonymized subjects who were students and professionals working and living near the PRN (*N* = 792), and evaluated our initial values for the branching logic version based off of the non-clinical means and standard deviations. Closing thresholds were first set at the 70th percentile for each subscale score. Opening triggers were set at the non-clinical item mean (since triggers are single-items) rounded down to the nearest whole number. These values were empirical guesses, which we knew we would re-evaluate with more data.

##### Act: branching logic fine tuning and digital deployment

**Fig. 2:**
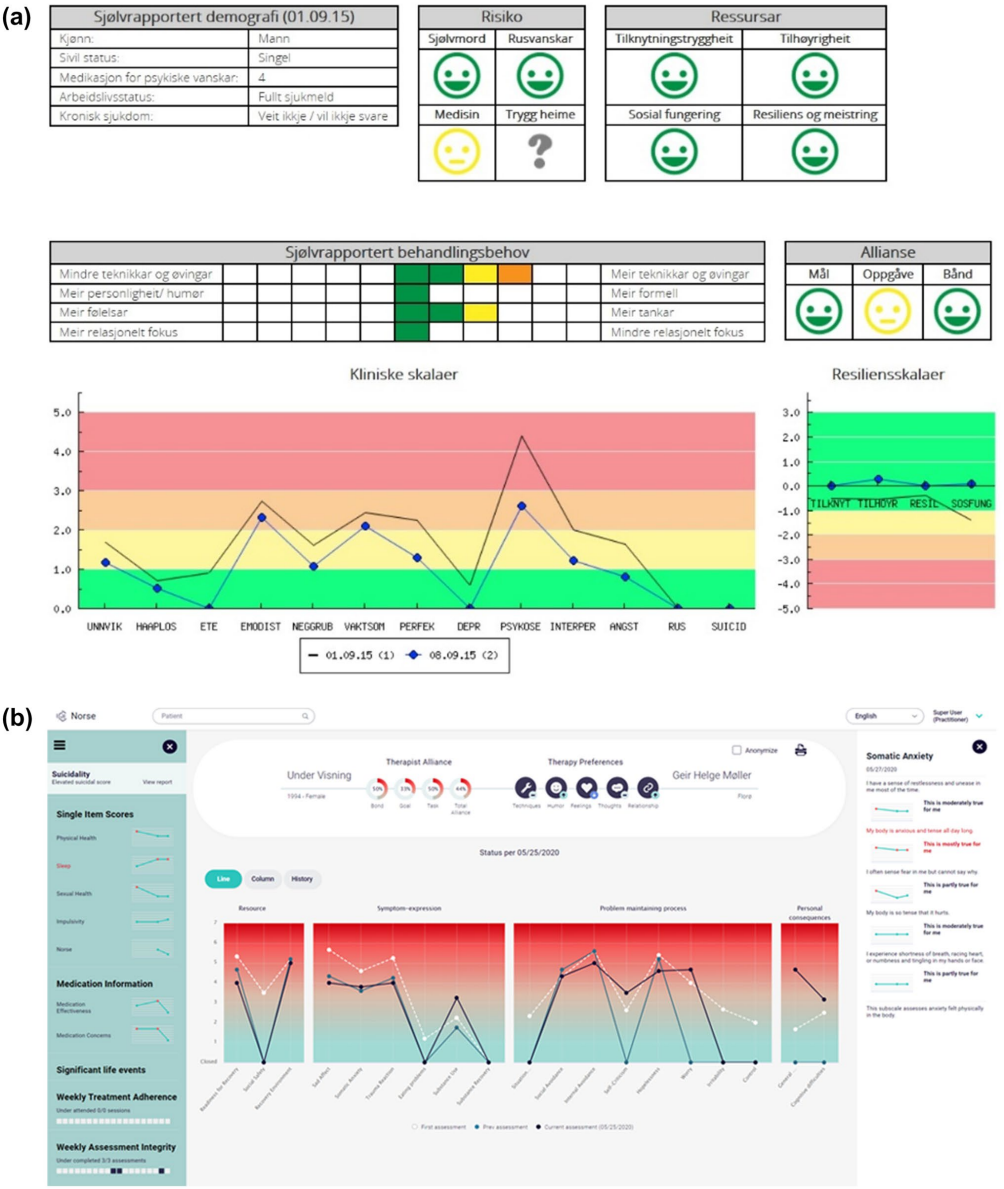
**a** Norse feedback software display after Cycle 2. Note: we provide several translations from Norwegian abbreviations to English below: Risiko-Risk; Ressursar-Single-Item Scores; Sjolvrapportert behandlingsbehov-Therapy-Feedback; Allianse-Alliance; Kliniske skaler-Clinical Scales; Resiliensskalaer-Strengths Scales; Unnvik-Avoidance; Haaplos-Demoralization; Ete-Eating Problems; Emodist-Emotional Distancing; Neggrub-Hurtful Rumination; Vaktsom-Hypervigilance; Perfek-Perfectionism; Depr-Depression; Psykose-Psychosis; Interper-Relational Distress; Angst-Somatic Anxiety; Rus-Substance Use; Suicid-Suicide Risk; Tilknyt-Attachment; Tilhoyr-Connectedness; Resil-Resilience and Personal Coping; Sosfung-Social Role Functioning. **b** Norse Feedback Software Display after Cycle 4

At this stage, the oversight group determined that the psychometrics and branching logic for the measure were good enough to test in the PRN, where routine outcomes monitoring and feedback had not yet been implemented. Our partner clinic was willing to deploy the measure to two inpatient units and one outpatient clinic (80 total clinical staff comprised of MD, PhD, and Nurse clinicians), and a new member of the oversight group was recruited—a mental health nurse with extensive experience within the health system, who could act as an implementation specialist and direct clinical support to our PRN partners. The dynamic assessment structure was transferred into the software system and the feedback format modified to reflect the changes (see Fig. 2a and b). Feedback was entirely digital—provided on-screen through the partner’s software system.

#### Third PDSA cycle (September 2015–September 2017)

##### Plan: re-evaluating items

This cycle reflects the first iteration of the full method we had hoped to deliver on—with strong quantitative processes and deep stakeholder feedback and involvement. We strongly suspected that the 1.0 version contained many items and some scales that were performing poorly, and focused the third cycle on using our clinical stakeholders alongside research data to evaluate the need to overhaul the measure. The second author was invited onto the oversight group at this point, to provide additional statistical and measure development expertise. To round out the oversight group, a clinical psychologist from one of the PRN sites, already practicing with NF [18] was brought into the oversight group as a qualitative research assistant and expert on the clinical use of the system. At this point, the oversight group consisted of four clinical psychologists and one psychiatric nurse, representing three universities and one practice clinic.

##### Decision to emphasize item response theory (IRT)

At this point in the development, we had an important methodological decision-point. The structure of the NF assessment was that of many small scales related to diverse phenomena of interest to stakeholders, and each scale was intended to operate independently. We needed to provide item-level feedback to our stakeholders, to assess whether to change or eliminate underperforming items. Therefore, we determined to adopt IRT methods as our principle means for further quantitative evaluation and development. The choice of this method is described in detail in McAleavey et al., [15].

##### Qualitative interview format

The oversight group elected to conduct focus groups with practitioners who had experience using the Norse Feedback system, and interviews with patients who had similar experience. We also determined this work should follow the quantitative analyses, so that results could be presented to the stakeholders for their feedback. This was particularly important, we determined, because we anticipated the need to re-word or replace many items from the measure, and wanted to avail ourselves of stakeholder expertise.

##### Do: data collection

Data for the third development cycle consisted of the 794 full administrations to anonymized volunteer participants collected as part of Cycle 2, and new data on 222 unique patients in inpatient and outpatient settings, collected as part of routine care within the PRN and anonymized before being provided to the authors for analysis (see Table 1). All clinical respondents completed the full 1.0 intake assessment. Of these, 140 completed at least one follow up assessment—the first follow up data we had collected.

##### Study: quantitative and qualitative findings

Specific results are presented elsewhere [15], and we focus here on how those results informed the method.

##### Quantitative results

Overall, while some scales captured ‘good enough’ information about the phenomena of interest, IRT analyses indicated ample room for improvement. When evaluating individual items, 42 items across the 17 subscales provided redundant or negligible information. The poorly performing items were marked for evaluation and revision so that stakeholders could give feedback on whether they thought the statistical findings mirrored their experience.

##### Qualitative results

The results of the quantitative analyses were evaluated in two stages: first, the oversight group met for three days to review the quantitative feedback and consider recommended solutions to present to a stakeholder feedback forum. During this process, the oversight group determined to break NF subscales into domains, to help with interpretation. Four domains were identified for ratification by the stakeholders: Symptom Expression (observed symptoms), Maintaining Processes (underlying mechanisms that drove symptoms), Resources (e.g. social supports, environment), and Personal Consequences (functional impairments). These recommendations were then presented to a group of eight practitioners who had been using the 1.0 version for at least a year, in a two-day event.

Each item that had been identified as underperforming was presented to the members of the feedback forum, alongside basic education on how to understand item level information curves. Participants provided their feedback on whether or not a scale or item had been clinically useful to them. Then, members of the forum made suggestions for items to replace those which had been eliminated, and suggestions for items or scales that had not been considered in the initial development. For example, practitioners collectively agreed that a scale measuring substance use habits, while effective at identifying how severe a patient’s substance use was, did not have the necessary sensitivity to track progress toward sobriety. They noted that much of the progress toward sobriety was related to developing coping skills and preparing to change substance use habits. Thus, they recommended the creation of a “substance recovery” scale, which would operate alongside the substance use scale and measure coping and preparedness, which signaled changes. They developed several candidate items for the scale, which were then reviewed and selected by the oversight group, which also determined that this scale would only be opened if a patient was elevated on the existing substance use scale.

This procedure was also purposively implemented in patient interviews with participants who had lived experience with specific items and scales. For example, the eating concerns scale analyses established that we needed a new high difficulty item. Patients with lived experiences with anorexia were able to suggest specific formulations to this end. This process, in sum, resulted in the creation of 52 new or modified items, for a total of 86 scale items, spread over 20 clinical scales, plus five single items, four alliance items, five therapy needs items and two medication feedback questions (Table 2).

##### Branching logic measure reduction

We also used the clinical repeated-measures data from this cycle to evaluate whether the measure was dynamically reducing in length as we hoped. For the 140 patients with more than one administration, the average number of items on the second administration dropped from 86 to 64, and the time to complete dropped from 14 to 8 min. By session five, the average dropped to 54. We concluded that the branching logic method we had selected appeared to be working, and we needed to refine how we determined the closing and opening values. We also committed to dedicating future cycles to evaluating even more patient-specific dynamic assessment methodology (although this is not in scope for the current paper).

##### Act: re-deployment of the new measure

In partnership with our software vendor, we prepared a new version—2.0—for deployment into clinical settings. Once completed, each of our clinical sites set a date to switch over to the 2.0 version from 1.0. Notably, because so many items and scales had changed, we discarded old norms, and reported raw scores only on the 2.0 version. We also had to re-estimate scale closing and opening logic based on the items that remained from 1.0. Our intention was to revise and update these as soon as possible, once enough data had been collected through our PRN, which had grown in Norway, with the addition of two more outpatient clinics and another 30 clinicians.

#### Fourth PDSA cycle (September 2017–June 2018)

##### Plan: re-evaluating branching logic

Our next plan phase was focused on rapidly re-evaluating our trigger items and opening/closing scale logic. We also re-evaluated our statistical process for determining which items should be used as triggers, and what our thresholds should be. In the last phase of development, the first with a clinical deployment, we learned from clinicians that NORSE could be overwhelming if too many scales appeared to be relevant. For example, a patient who is above the closing threshold on fourteen subscales represents a remarkably complex case conceptualization. Therefore, as part of our re-evaluation of the dynamic logic, we attempted to favor closing scales (increasing specificity), in order to allow clinicians to focus on the most critical and elevated.

##### Do: collecting PRN data

With our expanded PRN, data collection was more rapid, which allowed the fourth cycle to also be our shortest. We used full NORSE initial assessments from 323 patients in routine care at two of our PRN outpatient centers to evaluate triggers and thresholds. These data were de-identified, and provided for analysis without a codebook, so that participants’ identities could not be traced by the research team.

##### Study: methods for trigger item and thresholds

To establish trigger items and thresholds, we needed to identify which specific response value of the 7-point Likert scale, on which item, would be used to open the full scale for assessment, for each scale. First, we re-evaluated the criteria for our branching system by examining the reporting patterns on each scale and item (including response frequency histograms, average scale scores for clinical and non-clinical populations, the distribution, modality, and skew of scores). The oversight group recognized that only a subset of clinical patients, generally those with notably above-average scores, should be expected to receive clinical benefit by detailed assessment and tracking of that scale. The size and composition of this subset likely varies across scales. Items on the somatic anxiety scale were endorsed by significantly more patients than items on the substance use scale, for example, likely because anxiety is one of the most common experiences in mental health settings, while problematic substance use is often treated in specialized settings. Thus, some subscale-specific decisions were needed. Trigger thresholds which would open a closed scale were required to be at least greater than the clinical sample mean, and at a level where roughly 1/3 of the clinical sample was above the threshold. For some common problems (e.g., sad affect, somatic anxiety) the oversight group selected a threshold allowing for more individuals to complete the full scale, and for some less common concerns in typical settings (e.g., eating problems), this was expected to be lower. Such determinations were based on base rates in primary mental health settings and anticipated locations of the system’s use. This step established the location on the latent traits we wished to discriminate with most accuracy.

While we could have relied exclusively on observed frequencies of responses, we also incorporated IRT methods to provide model-based assessments for key components. Specifically, we estimated graded-response models [19] for each scale in order to evaluate which item on each scale had maximum information at the relevant level of severity (determined previously per scale). We then evaluated the predictive power of that item’s different response values to estimate, in isolation from other item responses, the total scale score. The item response value with the optimal sensitivity and specificity for discriminating full scale scores above or below the target was selected as the threshold for scale opening. This resulted in the revision of 9 of the initial 17 triggers, and the creation of 3 new trigger items for the new scales. Trigger opening values were changed for all 20 triggers, based on the new method (Table 3).

**Table 3:** Trigger item properties after PDSA Cycle Four, predicting whether a scale should be open

Subscale	Sensitivity (%)	Specificity (%)	PPV (%)	NPV (%)	Accuracy (%)	% triggering
Cognitive problems	77	94	92	82	86	40
Control	61	90	85	70	76	34
Eating	72	95	89	85	86	29
General functioning	66	85	83	70	75	24
Hopelessness	78	88	86	81	83	43
Internal avoidance	67	92	88	77	81	33
Irritability	48	98	90	85	86	13
Readiness for recovery	66	92	89	74	79	35
Recovery environment	71	82	69	84	78	33
Sad affect	86	89	87	87	87	47
Self-criticism	73	89	82	83	82	36
Situational avoidance	49	98	96	66	73	25
Social avoidance	71	96	95	79	84	35
Social safety	66	85	84	98	75	42
Somatic anxiety	79	83	81	81	81	46
Substance recovery	84	99	97	95	95	19
Substance use	84	99	97	95	95	19
Suicide	74	93	86	86	86	31
Trauma	61	95	91	76	90	29
Worry	82	91	93	77	85	52

*PPV* Positive predictive value, *NPV* negative predictive value; *% triggering* the % of patients who would receive the full scale using the current trigger value. The Substance Recovery scale has the same opening trigger as the Substance Use scale by design. It is only relevant when there is a substance use problem

A scale’s closing threshold is conceptualized as the value that best determines when that scale is no longer a pressing clinical concern. The major potential problem with these values is that if they are set too high, the scale may close prematurely, depriving clinicians of important clinical information on their treatment targets. At the time of initial development, we decided that we did not have sufficient evidence on which to base such determinations beyond our pre-existing rational process. Therefore, we adopted a conservative approach, using a three-session moving average set at two scale points lower than the opening thresholds for the commonly-endorsed scales. This prevents repetitively opening and closing the scale when a symptom is stable and requires a three-session moving average notably below a patient’s earlier level prior to closure. We included some exceptions for the less-commonly relevant scales, which have higher closing thresholds of only one scale point below the opening threshold. For high-risk scales (Substance Use and Suicide Risk), the opening threshold was almost as low as possible, so the closing threshold was simply set to be equal to this opening threshold. This helps maintain these scales in full when they have been previously relevant for a patient, until their severity is greatly diminished. Full evaluation of the empirically performance of these rationally-developed triggers is planned in future cycles. Relevant evidence to weigh includes patient and clinician experience, stability of scores over time in treatment, and predictive accuracy of the closing thresholds at different values.

##### Act: implementation into existing software

In collaboration with developers at the software partner, the new trigger items, opening thresholds, and closing values were incorporated into the NF assessment platform. Each participating member of the PRN was provided with advance warning, a “go-live” date, and training and support related to the clinical implications of the new branching logic.

#### Fifth PDSA cycle (June 2018–present)

The fifth PDSA cycle remains ongoing at the present writing, with the most recent data analysis and stakeholder feedback having just been completed in October, 2019. It is too early to report on the totality of these results, but worth noting that the cycle has been sustained and the quantitative results are presented in this issue [15]. These will shortly be followed by another stakeholder working group or groups.

In this cycle, we also began to sell the NF system to healthcare organizations within Norway. Our decision was grounded in three principle factors: (1) we needed revenue to build new software (see Fig. 2a and b) we believed was required to deliver the patient and clinician experiences we wanted; (2) knowing grants would not fund development forever, we needed to create a revenue stream that would support ongoing development; (3) implementation and training requirements for the effective clinical use of NF was time-consuming and expensive—we decided we needed to ask customers to pay for that service. The benefits of commercialization were weighed against the cost—the risk of bias entering our research process. We determined the need to bring in more transparency via publication, and the pull in more unbiased feedback and research-affiliates, to offset the potential for bias.

### Summary

Over the course of five years, NF development has tested a method for continuous development and stakeholder feedback, which has resulted in a CFS unlike others, and a supporting PRN and research infrastructure committed to further development. Involving patients and providers in the development process was, and remains, vital to the creation of NF. As we illustrated, we were confronted with several critical decision-points due to stakeholder feedback which have shaped NF into its current incarnation.

#### What worked?

In the NF project, knowing that the research and development processes were ongoing was freeing for us as researchers. We were able to rely on the knowledge of future PDSA cycles to move through the initial measure development process relatively quickly, i.e., without exhaustively selecting items or forming an item pool far larger than what we would ultimately use. This freeing feeling also applied to decisions related to the dynamic structure of the measure. We were relatively easily able to determine the method that we would like to test first, knowing that we would evaluate its effectiveness and adjust accordingly in later cycles.

From a practical perspective, the iteration from one cycle to the next allowed us to revisit our priorities and needs based on developments during the preceding cycle. This allowed for an almost complete shift in focus for the second cycle to developing a dynamic assessment methodology for administration. Similarly, our choice to embrace IRT was a methodological pivot, which had its genesis in the feedback from patients and providers: they wanted measures of diverse phenomena, not diagnoses. In short, knowing that there will be future opportunities to test ideas allows a research team to prioritize what to address in the immediate future and to flexibly change priorities based on feedback.

Lastly, the cyclical nature of the process freed us from feelings of defensiveness in the face of critical feedback—in fact, we went out of our way to solicit criticisms that would elicit improvement. Knowing that the system was never “done” allowed us to openly explore whether a decision had been a mistake, or a subscale needed to be changed or eliminated. This is not to say it was easy—each item and subscale had its champions—but it created, and continues to support, an academic hunger for criticism.

#### What did not work?

One immediate challenge behind the iterative nature of the work was that we did not have a good answer to reasonable requests for “a validation paper”. This made it more difficult for us to easily communicate the psychometric properties of NF to a potential new user. Similarly, as the system went through iterative change, we had to re-explain the scales and implementation to existing users.

Often, we made choices quickly when taking more time might have resulted in a better decision. Yearly cycles come surprisingly fast, and often we lacked the data necessary to make critical choices. This was readily evident in our challenge setting closing thresholds for redesigned subscales in Cycle 4. With another six months of data, we could have made a data-informed decision instead of a rational-informed one. However, because the system was already deployed to users, we had to decide between rolling out a much needed but incomplete update and holding the new version until we had more data.

Lastly, there is a risk of including too much in a system, to accommodate as much feedback as possible. We are not certain whether we have already crossed that threshold, but it remains a constant tension. Future challenges for this method will be determining how and whether to include adjustments requested for sizable minorities (e.g. including a borderline identity scale, or obsessive traits scale) as we have already done, for example, for eating disorders. This tension will further challenge our ability to build a system with utility for stakeholders while maintaining parsimony.

#### Conclusion

The intention of this method is to continue iterating in perpetuity—refining the system, its constituent measures, the software delivery system, and all the associated training and implementation, for years to come. To-date, the method has produced a system that is different, both in structure and (to degrees) in content, that has fair to good psychometric properties [15], and that is closely tied to the clinical practices involved with the feedback system. Whether this method produces a CFS that serves stakeholders better than the current offerings is a matter for future evaluation.

## References

[1] Beidas RS, Stewart RE, Walsh L, Lucas S, Downey MM, Jackson K (2015). Free, brief, and validated: Standardized instruments for low-resource mental health settings. Cognitive and Behavioral Practice.

[2] Smith GT, McCarthy DM (1995). Methodological considerations in the refinement of clinical assessment instruments. Psychological Assessment.

[3] Tarescavage AM, Ben-Porath YS (2014). Psychotherapeutic outcomes measures: A critical review for practitioners. Journal of Clinical Psychology.

[4] Youngstrom EA, Van Meter A, Frazier TW, Hunsley J, Prinstein MJ, Ong M-L, Youngstrom JK (2017). Evidence-based assessment as an integrative model for applying psychological science to guide the voyage of treatment. Clinical Psychology: Science and Practice.

[5] Nordberg, S. S., McAleavey, A. A., Solstad, S., & Moltu, C. (*in submission*). Three methodological obstacles for clinical feedback systems: Updating methods by continuous improvement.

[6] Sales CM, Neves IT, Alves PG, Ashworth M (2018). Capturing and missing the patient's story through outcome measures: A thematic comparison of patient-generated items in PSYCHLOPS with CORE-OM and PHQ-9. Health Expectations.

[7] Cronbach LJ, Meehl PE (1955). Construct validity in psychological tests. Psychological Bulletin.

[8] Hodach R, Grundy P, Jain A (2016). Provider-led population health management: Key healthcare strategies in the cognitive era.

[9] Toussaint J, Gerard RA (2010). On the mend: Revolutionizing healthcare to save lives and transform the industry.

[10] Brydon-Miller M, Greenwood D, Maguire P (2003). Why action research?. Action Research.

[11] Castonguay LG, Barkham M, Lutz W, McAleavey AA, Lambert MJ (2013). Practice-oriented research: Approaches and application. Bergin and Garfield’s handbook of psychotherapy and behavior change.

[12] Bickman L, Kelley SD, Athay M (2012). The technology of measurement feedback systems. Couple & Family Psychology.

[13] Moltu C, Veseth M, Stefansen J, Nøtnes JC, Skjølberg Å, Binder PE (2018). This is what I need a clinical feedback system to do for me: A qualitative inquiry into therapists’ and patients’ perspectives. Psychotherapy research.

[14] Moltu C, Stefansen J, Nøtnes JC, Skjølberg Å, Veseth M (2017). What are “good outcomes” in public mental health settings? A qualitative exploration of clients’ and therapists’ experiences. International Journal of Mental Health Systems.

[15] McAleavey, A. A., Nordberg, S. S., & Moltu, C. (under review) Initial quantitative development of the Norse Feedback system: A novel adaptive multidimensional tool for use in routine mental healthcare 10.1007/s11136-021-02825-1 PMC8528796 33851326

[16] Hovland, R., & Moltu, C. (2019, in press). Making way for a clinical feedback system in the narrow space between sessions: Navigating competing demands in complex healthcare settings. International *Journal of Mental Health Systems*10.1186/s13033-019-0324-5 PMC6825345 31700530

[17] Hovland R, Moltu C (2019). The challenges of making clinical feedback in psychotherapy benefit all users: A qualitative study. Nordic Psychology.

[18] Helleseth, M., Nordberg, S. S., McAleavey, A. A., & Moltu, C. (2018) A clinician's experience with using NORSE for routine outcome monitoring and feedback in ordinary out-patient practice: two clinical case examples. Presented at the Annual Meeting of the Society for Psychotherapy Research, Amsterdam, Netherlands, June 27–30, 2018.

[19] Samejima F (1969). Estimation of latent ability using a response pattern of graded scores. Psychometrika Monograph Supplement.

